# Lung cancer diagnosis and staging with endobronchial ultrasound-guided transbronchial needle aspiration compared with conventional approaches: an open-label, pragmatic, randomised controlled trial

**DOI:** 10.1016/S2213-2600(15)00029-6

**Published:** 2015-04

**Authors:** Neal Navani, Matthew Nankivell, David R Lawrence, Sara Lock, Himender Makker, David R Baldwin, Richard J Stephens, Mahesh K Parmar, Stephen G Spiro, Stephen Morris, Sam M Janes, on behalf of the Lung-BOOST trial investigators

**Affiliations:** a Department of Thoracic Medicine, University College London Hospital, London, UK; b Lungs for Living Research Centre, UCL Respiratory, University College London, London, UK; c Medical Research Council Clinical Trials Unit, London, UK; d Department of Cardiothoracic Surgery, The Heart Hospital, London, UK; e Department of Respiratory Medicine, Whittington Hospital, London, UK; f Department of Respiratory Medicine, North Middlesex University Hospital, London, UK; g Department of Respiratory Medicine, Nottingham University Hospital, Nottingham, UK; h Department of Respiratory Medicine, Royal Brompton Hospital, London, UK; i Department of Applied Health Research, University College London, London, UK

## Abstract

**Background:**

The diagnosis and staging of lung cancer is an important process that identifies treatment options and guides disease prognosis. We aimed to assess endobronchial ultrasound-guided transbronchial needle aspiration as an initial investigation technique for patients with suspected lung cancer.

**Methods:**

In this open-label, multicentre, pragmatic, randomised controlled trial, we recruited patients who had undergone a CT scan and had suspected stage I to IIIA lung cancer, from six UK centres and randomly assigned them to either endobronchial ultrasound-guided transbronchial needle aspiration (EBUS-TBNA) or conventional diagnosis and staging (CDS), for further investigation and staging. If a target node could not be accessed by EBUS-TBNA, then endoscopic ultrasound-guided fine needle aspiration (EUS-FNA) was allowed as an alternative procedure. Randomisation was stratified according to the presence of mediastinal lymph nodes measuring 1 cm or more in the short axis and by recruiting centre. We used a telephone randomisation method with permuted blocks of four generated by a computer. Because of the nature of the intervention, masking of participants and consenting investigators was not possible. The primary endpoint was the time-to-treatment decision after completion of the diagnostic and staging investigations and analysis was by intention-to-diagnose. This trial is registered with ClinicalTrials.gov, number NCT00652769.

**Findings:**

Between June 10, 2008, and July 4, 2011, we randomly allocated 133 patients to treatment: 66 to EBUS-TBNA and 67 to CDS (one later withdrew consent). Two patients from the EBUS-TBNA group underwent EUS-FNA. The median time to treatment decision was shorter with EBUS-TBNA (14 days; 95% CI 14–15) than with CDS (29 days; 23–35) resulting in a hazard ratio of 1·98, (1·39–2·82, p<0·0001). One patient in each group had a pneumothorax from a CT-guided biopsy sample; the patient from the CDS group needed intercostal drainage and was admitted to hospital.

**Interpretation:**

Transbronchial needle aspiration guided by endobronchial ultrasound should be considered as the initial investigation for patients with suspected lung cancer, because it reduces the time to treatment decision compared with conventional diagnosis and staging techniques.

**Funding:**

UK Medical Research Council.

## Introduction

Lung cancer is the most common cause of cancer death across the world.^1^ The clinical staging of non-small-cell lung cancer is an important process that identifies treatment options and guides disease prognosis. In patients with non-small-cell lung cancer who are fit for surgery and have no evidence of extrathoracic spread, the disease status of the mediastinal lymph nodes can be used to establish a patient's suitability for treatment with curative intent.^2^, ^3^

Several invasive and non-invasive techniques are available to support the diagnosis and staging of lung cancer. Patients with suspected lung cancer undergo a CT scan of the lower neck, thorax, and upper abdomen. About 50% of patients present with metastatic disease that is evident outside the thorax^4^ and, in these patients, a biopsy sample taken from the safest most accessible location is recommended. However, in patients with solely intrathoracic disease evident on the initial CT scan, the diagnostic and staging algorithm is more complex. A sample of the primary lesion is generally taken by bronchoscopy or CT-guided biopsy before attention turns to mediastinal nodal staging. PET-CT is reliable if mediastinal lymph nodes that are less than 1 cm in the short axis are negative. However, invasive sampling of mediastinal lymphadenopathy is recommended when lymph nodes are avid for ^18^F-fluorodeoxyglucose (^18^F-FDG), the tumour is central, there is a PET-positive hilar lymph node, or any mediastinal node is larger than 1 cm in the short axis (irrespective of ^18^F-FDG uptake).^5^

The diagnosis and staging of patients with intrathoracic disease can therefore need several investigative procedures, including bronchoscopy, radiology-guided biopsy sampling, PET-CT, and mediastinoscopy. This process often takes several weeks and is a time of great anxiety for patients. Additionally, 26% of patients with lung cancer report that their health deteriorates while waiting for an hospital appointment.^6^ Further time will elapse before a treatment decision has been made which could mean that they are unfit for oncological treatments by the time a treatment decision has been reached.

The present approach to mediastinal staging of non-small-cell lung cancer (CT, PET-CT, and mediastinoscopy) can result in inaccurate nodal staging in 25% of operable patients,^7^ perhaps because the sensitivity for the detection of mediastinal metastases by CT scan is 51%, by PET-CT is 74%, and by mediastinoscopy is 78%.^5^, ^8^

Endobronchial ultrasound-guided transbronchial needle aspiration (EBUS-TBNA) is a newer technique that allows minimally invasive sampling of all intrathoracic lymph nodes adjacent to the bronchial tree. A pooled analysis of 1299 patients^9^ with known or suspected non-small-cell lung cancer undergoing EBUS-TBNA showed that the procedure had a sensitivity of 90% for the detection of mediastinal nodal metastases. At the time of the inception of our trial in 2007, guidelines^4^ recommended EBUS-TBNA as an alternative to mediastinoscopy for patients who needed invasive mediastinal sampling after a PET-CT scan. Invasive mediastinal sampling is also recommended for staging patients with central tumours or patients with enlarged or ^18^F-FDG-avid hilar lymphadenopathy.

Therefore we aimed to investigate whether EBUS-TBNA could be used as an initial investigation for the diagnosis and staging of patients with suspected lung cancer because the procedure provides a tissue diagnosis and nodal staging in one investigation. Previous studies have shown that EBUS-TBNA might represent good value for money,^10^, ^11^ but there is a shortage of information about its efficacy or cost-effectiveness for patients with suspected lung cancer. We therefore did the Lung-BOOST (BronchOscopic or Oesophageal ultrasound for lung cancer diagnosis and STaging) trial—a pragmatic, multicentre, randomised controlled trial to test the hypothesis that EBUS-TBNA as an initial investigation after a staging CT scan would reduce the time to treatment decision, and reduce the number of investigations needed for the diagnosis and staging of patients with suspected lung cancer at no additional cost compared with conventional diagnosis and staging (CDS) techniques.

## Methods

### Study design and participants

We did this randomised controlled trial in six centres in the UK (University College London Hospital, Whittington Hospital, North Middlesex University Hospital, Princess Alexandra Hospital, Barnet General Hospital, and Nottingham University Hospital). Patients at these centres who were suspected to have stage I to IIIA lung cancer on the basis of CT scans of the neck, thorax, and upper abdomen were eligible for trial entry. For inclusion into the trial, patients had to be aged at least 18 years and fit enough to undergo thoracotomy and lung resection. Exclusion criteria were significant concurrent malignant disease or any condition or concurrent medicine that contraindicated EBUS-TBNA or mediastinoscopy. Patients with known extrathoracic malignant disease, supraclavicular lymphadenopathy, or pleural effusion were also excluded. The 7th edition of the tumour, node, metastasis (TNM) staging system in lung cancer was used throughout.^12^

This investigator-initiated pragmatic trial was approved by the UK national research ethics service (reference 07/H0711/127) and the ethics committees of the six participating centres. Patients provided written informed consent.

### Randomisation and masking

We randomly assigned participants (1:1) to either conventional diagnosis and staging (CDS group) or EBUS-TBNA as an initial investigation after a staging CT scan followed by further diagnosis and staging techniques if needed (EBUS group). We used a telephone randomisation method with permuted computer-generated blocks of four. Randomisation was stratified according to the presence of mediastinal lymph nodes that measured 1 cm or more in the short axis and by recruiting centre. An investigator undertook the informed consent process, followed by the telephone randomisation process done by research assistants. The random allocation sequence was kept in the randomisation centre and concealed from participants and investigators until the interventions were assigned. Because of the nature of the intervention, masking of participants and consenting investigators was not possible. However, pathologists and radiologists were unaware that patients were enrolled into a clinical trial. Data were obtained on paper-based case forms and entered by an independent clerk onto a secured trial database on a dedicated trial computer.

### Procedures

Participants allocated to CDS underwent investigations as determined by the local multidisciplinary team. We suggested an algorithm for CDS in the trial protocol based on the most recently available UK National Institute of Health and Clinical Excellence guidance (2005)^13^ at the time the trial started (appendix). The trial management group agreed that allowing the responsible multidisciplinary teams to determine the patients' investigations would provide the best comparator group. This allowed the control CDS group to emulate clinical practice, giving the trial strong external validity. Patients randomly assigned to the EBUS group underwent EBUS-TBNA as an initial procedure after a staging CT scan (appendix). The procedure was done in the outpatient setting with patients given moderate sedation with midazolam and fentanyl. We did EBUS-TBNA using a dedicated bronchoscope with a linear ultrasound probe integrated into the distal end (BF-UC160F-OL8, Olympus, Tokyo, Japan, or EB 1970UK, Pentax, Slough, UK). A systematic examination of all mediastinal and hilar lymph node stations was made. Nodes that we suspected were metastatic because of their size or location on the CT scan were aspirated with a 22-gauge or 21-gauge needle and labelled according to the Mountain–Dressler lymph node map. If no enlarged nodes were identified, samples were taken via EBUS-TBNA from the lymph node station that was most likely to drain the primary lesion.^14^ If a target node was inaccessible with EBUS-TBNA then endoscopic ultrasound-guided fine needle aspiration (EUS-FNA) as an alternative procedure was allowed. After EBUS-TBNA, any further investigations needed were established by the multidisciplinary team.

Patients were given treatment according to the recommendations of the multidisciplinary team. PET-CT was recommended for all patients before a decision to treat with curative intent. In the absence of symptoms, we did not undertake surveillance for brain or bone metastases unless radical treatment was planned.

### Outcomes

The primary endpoint was the time from first outpatient appointment with the respiratory specialist to treatment decision by the multidisciplinary team, after completion of the diagnosis and staging procedures. We prespecified that the primary outcome measure would be analysed in the subgroup of patients with non-small-cell lung cancer. We prespecified four secondary endpoints: (1) UK National Health Service (NHS) costs of diagnosing and staging lung cancer, (2) the number of investigations and outpatient attendances per patient, (3) the proportion of patients diagnosed and staged with one procedure, and (4) the number of avoidable thoracotomies. An avoidable thoracotomy was defined as an open and close procedure, unexpected mediastinal nodal metastases (pN2/pN3), pT4 or pM1a/b disease, resection of benign disease or disease recurrence, or death within 1 year of thoracotomy. We also documented the sensitivity, negative predictive value, and diagnostic accuracy of EBUS-TBNA a priori, and overall survival (post-hoc analysis) and complications from the different diagnostic and staging techniques.

The incremental cost of EBUS-TBNA as an initial investigation compared with CDS was calculated from the perspective of the NHS. We included the costs associated with all diagnostic and staging investigations. We also calculated the costs of treating patients diagnosed with lung cancer. Resource use data were obtained prospectively in the trial. Unit costs were obtained from NHS reference costs,^15^ NICE 2011 lung cancer guideline,^2^ and a published study;^10^ these were multiplied by the resource use and summed across all resource items.

### Statistical analysis

A priori, we expected that 80% of patients would be diagnosed and staged with only one investigation in the EBUS group, compared with 33% in the CDS group. Before the trial began, clinical practice was assessed in a retrospective analysis of diagnostic and staging procedures in five of the participating centres (data not shown). On the basis of this analysis, we estimated that patients in the CDS group of the trial would need a median time to treatment decision of 30 days, and patients in the EBUS group would need a median of 14 days. A sample size of at least 130 patients was planned to give 99% power, assuming a type 1 error of 5%.

Analyses were done for the intention-to-diagnose population. The Kaplan-Meier method was used to analyse the primary endpoint (time-to-treatment decision). Hazard ratios (HRs) were calculated from a Cox model, and did not include adjustment for any baseline factors. We used standard definitions of sensitivity for the detection of nodal metastases. The final diagnosis of nodal staging was established in both groups by clinical follow-up of at least 1 year and pathological changes noted with EBUS-TBNA, conventional TBNA, EUS-FNA, mediastinoscopy, or dissection of mediastinal lymph nodes.

The Fisher exact test was used to analyse categorical data, and unpaired *t* tests were used to compare groups of continuous normally distributed variables. All tests were two-sided and 5% was taken as the cutoff for statistical significance. The normal approximation method was used to calculate confidence intervals for the proportions. Final statistical analyses were done with STATA (version 10).

This trial is registered on ClinicalTrials.gov, number NCT00652769.

### Role of the funding source

The funders of the study had no role in study design, data collection, data analysis, or writing of the report. The corresponding author had full access to all the data in the study and had final responsibility for the decision to submit for publication.

## Results

**Figure 1:**
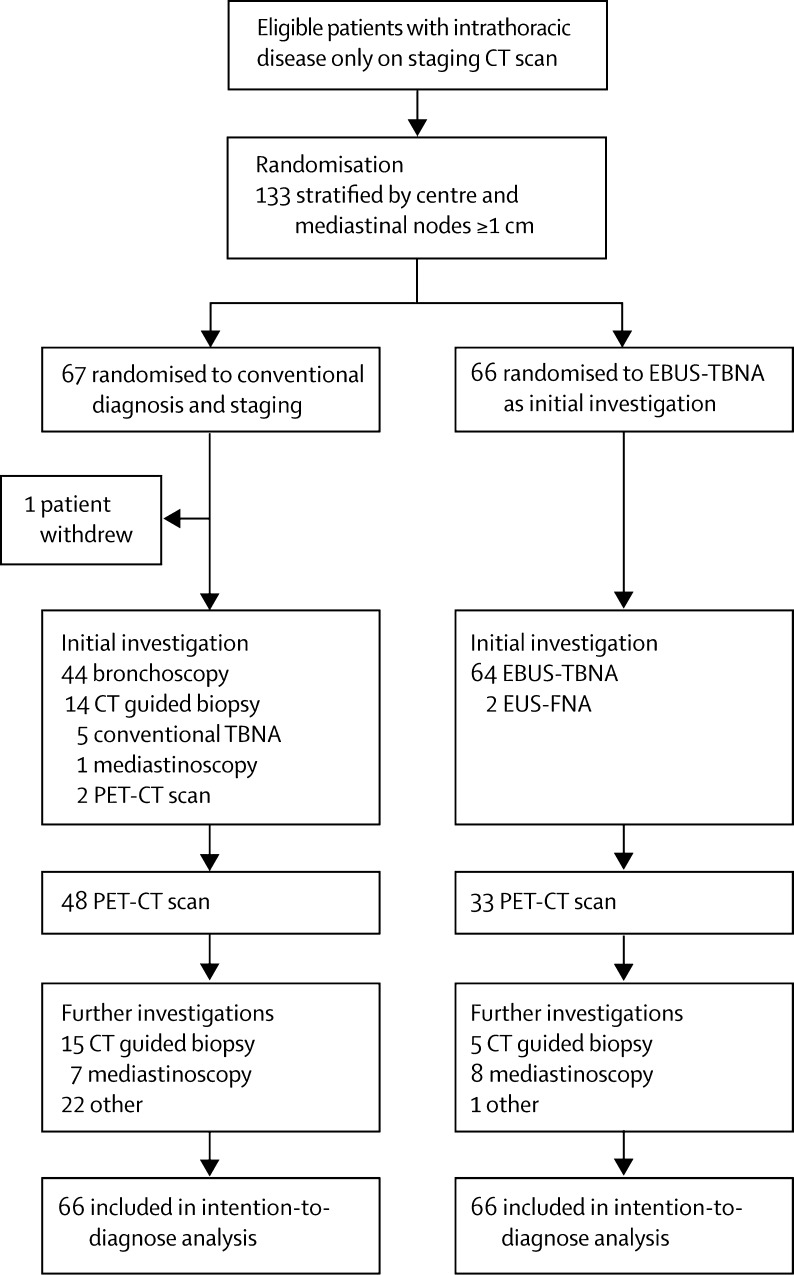
Trial profile EBUS-TBNA=endobronchial ultrasound-guided transbronchial needle aspiration. EUS-FNA=endoscopic ultrasound-guided fine needle aspiration. NSCLC=non-small-cell lung cancer.

Between June 10, 2008, and July 4, 2011, we randomly assigned 133 patients with suspected lung cancer to the CDS group (n=67) and EBUS group (n=66) (figure 1). One patient (randomly assigned to CDS) declined all further investigations and withdrew consent before any investigations were done. Both groups were well balanced for all major clinical characteristics (table 1).

Lung cancer was diagnosed in 57 (86%) patients in the CDS group and 50 (76%) in the EBUS group (p=0·196), and clinical staging did not differ significantly between the groups in patients with non-small-cell lung cancer (table 2). The benign final diagnoses were pneumonia, organising pneumonia, lung abscess, and folded lung.

**Figure 2:**
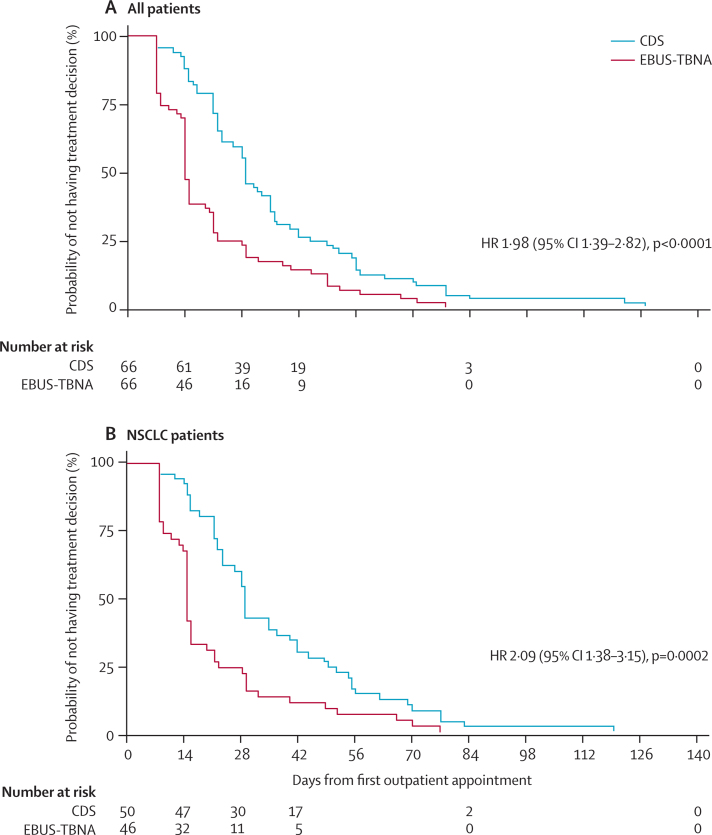
Time to treatment decision in all patients (A) and in those with non-small-cell lung cancer (B) Kaplan-Meier plots for (A) all patients and (B) patients with non-small-cell lung cancer only undergoing CDS or EBUS-TBNA. CDS=conventional diagnosis and staging. NSCLC=non-small-cell lung cancer. EBUS-TBNA=endobronchial ultrasound-guided transbronchial needle aspiration. HR=hazard ratio.

The median time-to-treatment decision was longer after CDS (29 days [95% CI 23–35]), than after EBUS (14 days [14–15]; HR 1·98, 95% CI 1·39–2·82, p<0·0001) in the intention-to-diagnose population. Therefore patients in the EBUS group of the trial were likely to receive a treatment decision twice as fast as patients in the CDS group (figure 2A). A greater proportion of patients had diagnosis and staging completed by 14 days in the EBUS group than in the CDS group (35 [53%] *vs* 8 [12%], p<0·0001). In the subset of patients with non-small-cell lung cancer (figure 2B), initial EBUS-TBNA resulted in a shorter time-to-treatment decision of 15 days (95% CI 14–16), compared with 30 days (95% CI 23–34) in the CDS group (HR 2·09, 95% CI 1·38–3·15, p=0·0002).

The mean number of investigations per patient, PET scans (figure 1), and avoidable thoracotomies at 1 year (table 3). were all significantly lower in the EBUS group than in the CDS group, and the number of patients diagnosed and staged with one investigation was greater (table 3). There were fewer PET scans in the EBUS group (33 of 66 [50%]) than in the CDS group (48 of 66 [73%]; p=0·002); however, the number of patients having treatment with curative intent was similar in each group (appendix).

In the CDS group, 44 (67%) of 66 patients initially underwent a bronchoscopy and 29 (44%) had a radiology-guided biopsy sample taken; in the EBUS group, 64 (97%) of 66 underwent EBUS and two (3%) had EUS-FNA as an initial procedure. Five (8%) of 66 patients had a subsequent radiology-guided biopsy sample taken (appendix). The number of mediastinoscopies did not differ between groups.

**Figure 3:**
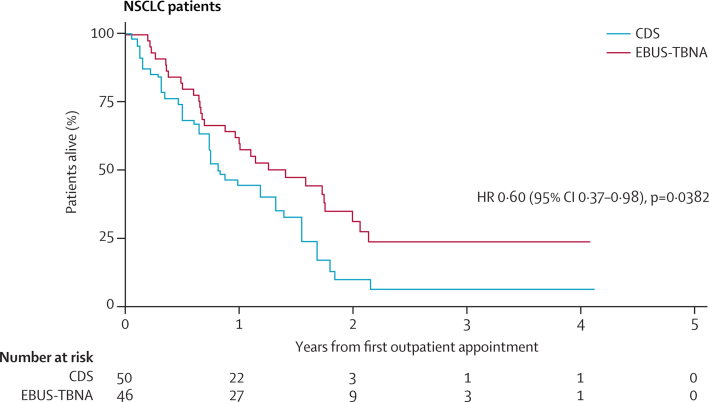
Overall survival of patients with non-small-cell lung cancer Survival of patients with non-small-cell lung cancer undergoing CDS or EBUS-TBNA. NSCLC=non-small-cell lung cancer. CDS=conventional diagnosis and staging. EBUS-TBNA=endobronchial ultrasound-guided transbronchial needle aspiration. HR=hazard ratio.

In a post-hoc analysis, the median survival of patients with non-small-cell lung cancer in the EBUS group of 503 days (95% CI 312–715) was longer than the median survival in the CDS group of 312 days (95% CI 231–488; HR 0·60, 0·37–0·98, p=0·0382; figure 3). An exploratory analysis of lung cancer patients who underwent surgery suggested that postoperative survival was better in the EBUS group than in the CDS group (appendix).

64 patients in the trial underwent EBUS-TBNA. The median size of lymph nodes sampled was 12 mm (IQR 7–20). The sensitivity of EBUS-TBNA in this trial was 92% (95% CI 78–98). The negative predictive value of EBUS-TBNA was 90% (72–97) and diagnostic accuracy was 95% (86–99). Two patients randomised to the EBUS group of the trial underwent EUS-FNA instead of EBUS-TBNA, both of whom had station 5 lymph nodes. The procedure yielded a diagnosis of malignant disease in both patients (one adenocarcinoma and one large cell lung cancer). In the CDS group of the study, five patients underwent conventional TBNA. Two of these patients had a benign final diagnosis, and in one patient conventional TBNA provided a diagnosis of squamous cell lung cancer. In the remaining two patients undergoing conventional TBNA, a negative procedure was followed by a mediastinoscopy that showed mediastinal metastases.

One patient in each group had a pneumothorax from a CT-guided biopsy sample; the patient in the CDS group needed intercostal drainage and was admitted to hospital.

The mean cost per patient for diagnostic and staging investigations was £2407 (SD £180·50) in the EBUS group and £2348 (192·20) in the CDS group (difference £59, 95% CI −£463 to £581; appendix). Mean initial treatment costs per patient in those diagnosed with lung cancer were £4452 (£180·00) and £4261 (£257·90), respectively (difference £191, 95% CI −447 to 829; appendix).

## Discussion

The results from our trial suggest that routine use of EBUS-TBNA as an initial investigation after a staging CT for suspected lung cancer scan results in a faster treatment decision, with fewer investigations at no significant difference in cost, and, in post-hoc analysis, seems to improve survival, compared with conventional diagnosis and staging methods (panel).

The primary endpoint of our Lung-BOOST trial was the time to treatment decision after the test, and the trial showed that routine and upfront use of EBUS-TBNA in the diagnostic pathway can reduce the median time-to-treatment decision from 29 days to 14 days. UK government initiatives in the NHS Cancer Plan have mandated since 2005 that patients receive treatment within 62 days of referral, with a maximum of 31 days between the decision to treat and the patient receiving treatment.^16^ The time that patients spend undergoing diagnostic and staging investigations is a time of great anxiety for patients, particularly because the median survival for all patients with lung cancer is poor (6·2 months). Importantly, 26% of patients self-report that their health deteriorates while awaiting a treatment decision.^6^ Therefore, the primary outcome measure in our trial of time-to-treatment decision is of great importance to patients and the multidisciplinary teams charged with their care. The results from the trial show that EBUS-TBNA can provide sufficient diagnostic and staging information in 45% of patients to define the treatment plan. PET-CT is recommended for all patients unless previous investigations have already shown that curative treatment is not an option. Many patients diagnosed with N2 disease by EBUS-TBNA will still need further investigations, including PET-CT scans if combination chemoradiotherapy or surgery are being considered. However, in this trial PET-CT was only needed for 19% of patients after a positive EBUS-TBNA. 25 patients with N2 disease in the EBUS group did not need a PET scan; of these, four could be candidates for chemoradiotherapy and might have a PET scan. Even if the wait for this scan took an extra week, this wait would not significantly affect the median time-to-treatment decision. Routine use of EBUS-TBNA was able to reduce time-to-treatment decision mainly by reducing the number of outpatient appointments and investigations (particularly PET-CT scans).

The Lung-BOOST trial recruited patients over 3 years, and the rate of accrual was similar to a randomised trial of PET-CT in lung cancer staging.^17^ Since trial inception, EBUS-TBNA has become an important investigation for patients with lung cancer and is now preferred to mediastinoscopy as an initial investigation for nodal staging. However, much of the data that lend support to its usefulness are based on case series, many of which are retrospective and therefore limited by selection bias. The randomised design of our trial reduces bias because the EBUS-TBNA operators could not choose patients for the procedure. Despite not being able to select patients, the sensitivity of EBUS-TBNA in the study was high. The introduction of EBUS-TBNA in practice has shifted the methods of tissue acquisition in patients with lung cancer from flexible bronchoscopy and radiology-guided biopsy sampling to EBUS-TBNA. However, to our knowledge, this is the first randomised trial to show the effect of EBUS-TBNA alone on clinical outcomes for patients with lung cancer.

The number of mediastinoscopies in the trial was low and did not differ between groups. However, the accuracy of preoperative mediastinal node staging was high, justifying the approach of the multidisciplinary teams. Only one patient in the trial had unexpected pathological N2 disease at thoracotomy and had previously undergone both EBUS and mediastinoscopy. Previous randomised trials of lung cancer staging have used mediastinoscopy routinely as part of clinical staging of lung cancer.^17^, ^18^ For example, ASTER^18^ showed that the combination of EUS with EBUS (followed by mediastinoscopy if EBUS or EUS was negative) was more effective than mediastinoscopy alone for diagnosis of mediastinal metastases. Our trial substantiates that mediastinoscopy is rarely needed for the preoperative staging of non-small-cell lung cancer in clinical practice. The results from this trial also suggest that EBUS-TBNA could be used as a primary diagnostic method in patients with suspected lung cancer rather than only as an alternative to mediastinoscopy.

In this trial, EBUS-TBNA was done by systematic assessment of all visible lymph node stations. Biopsy samples were taken from enlarged lymph nodes and lymph nodes that anatomically drained the lung cancer primary lesion. This approach achieved a sensitivity of 92% for nodal staging. Application of PET-CT, with 74% sensitivity for nodal staging,^5^ is therefore not needed before EBUS-TBNA. PET-CT, however, remains essential for the accurate systemic staging of non-small-cell lung cancer before radical treatment.

The management of advanced non-small-cell lung cancer now relies on phenotypic sub-classification and genotypic analysis of tumours. 41 patients (38%) were diagnosed with inoperable lung cancer by EBUS. Specimens obtained from EBUS-TBNA are accepted as suitable for the personalised approach to the management of non-small-cell lung cancer. In a multicentre study,^19^ specimens from EBUS-TBNA were suitable for EGFR mutation analysis in 90% of cases.

The results of the cost analysis suggested that use of EBUS-TBNA as an initial investigation after a CT scan was not more expensive than CDS. Because patients in the EBUS group of the trial had an earlier treatment decision (the primary outcome), we can conclude that EBUS-TBNA was more effective for the same cost, and was therefore cost-effective.

There were more avoidable thoracotomies at 1 year in the CDS group (13 [76%]) than in the EBUS group (5 [29%]). The broader definition of avoidable thoracotomy in this trial accounts for the high proportion compared with unnecessary thoracotomies in previous studies.^18^

In a post-hoc analysis, survival was higher in patients who underwent EBUS-TBNA as part of their diagnostic and staging strategy. Further post-hoc analysis suggests that this difference in survival might be due to superior selection (ie, not attributable to health at baseline) of candidates for surgery in patients undergoing preoperative EBUS-TBNA (appendix). We postulate that routine preoperative use of EBUS-TBNA and sampling of mediastinal lymph nodes that anatomically drain the primary tumour might result in a refined population undergoing surgery, with improved survival in that patient group. Because the use of EBUS-TBNA halved the time-to-treatment decision, earlier treatment, when the patient is fitter, could also improve outcome. The result of a survival advantage for patients undergoing EBUS needs to be replicated.

We recognise that the trial had several limitations. For example, the pragmatic nature of the trial meant that a consistent diagnostic and staging algorithm was not used across all the trial centres. However, the design of the study (which was undertaken at two teaching hospitals and four general hospitals) gives the results strong external validity. Advances in radiotherapy techniques during the trial period could mean that more patients with mediastinal metastases might now suitable for radical treatment and therefore would need PET-CT. In this pragmatic trial of patients with suspected lung cancer, 19% of the participants had a final diagnosis of disorders other than lung cancer, including metastatic melanoma and lung abscess. Despite this, the primary endpoint was statistically significant for both all patients and also those with non-small-cell lung cancer only. Finally, in this trial, EBUS-TBNA was undertaken by clinicians who were skilled in the procedure—the sensitivity of EBUS-TBNA might not be immediately reproducible in other centres.

In conclusion, when EBUS-TBNA is used as an initial investigation method after a CT scan in patients with suspected lung cancer confined to the thorax, it can provide a diagnosis and accurate nodal stage in one investigation. This results in a reduction in the time-to-treatment decision and might improve survival in patients with lung cancer when compared with a conventional diagnostic and staging strategy, at no additional cost.

## Figures and Tables

**Table 1:** Baseline characteristics

		**Conventional diagnosis and staging (n=66)**	**Endobronchial ultrasound-guided transbronchial needle aspiration (n=66)**
Age (years)		68 (IQR 61–73)	71 (IQR 62–78)
Men		46 (70%)	43 (65%)
Women		20 (30%)	23 (35%)
Ethnic origin			
	White	59 (89%)	51 (77%)
	Asian	2 (3%)	6 (9%)
	African	2 (3%)	4 (6%)
	Caribbean	2 (3%)	3 (5%)
	Other	1 (2%)	2 (3%)
ECOG performance status 0 or 1		57 (96%)	60 (92%)
Pack-years smoking history		42 (23·4)	42 (28·1)
FEV_1_ (L)		1·9 (0·72)	1·9 (0·65)
Clinical nodal staging on initial CT scan			
	N0	20 (30%)	21 (32%)
	N1	9 (14%)	6 (9%)
	N2	33 (50%)	34 (51%)
	N3	4 (6%)	5 (8%)

Data are median (range, IQR), n (%), or mean (SD), unless otherwise stated. FEV_1_=forced expiratory volume in 1 s. ECOG=Eastern Cooperative Oncology Group.

**Table 2:** Final diagnoses and stages of non-small-cell lung cancer

		**Conventional diagnosis and staging (n=66)**	**Endobronchial ultrasound-guided transbronchial needle aspiration (n=66)**
Benign lesion		6 (9%)	14 (21%)
Extrathoracic malignancy		3 (5%)	2 (3%)
Small cell lung cancer		7 (11%)	4 (6%)
Non-small-cell lung cancer		50 (76%)	46 (70%)
	Adenocarcinoma	21 (42%)	26 (57%)
	Squamous cell	21 (42%)	17 (37%)
	Large cell	3 (6%)	1 (2%)
	Adenosquamous	2 (4%)	1 (2%)
	Not otherwise specified	3 (6%)	1 (2%)
Stage IA/B		11 (22%)	10 (22%)
Stage IIA/B		10 (20%)	6 (13%)
Stage IIIA		20 (40%)	22 (48%)
Stage IIIB		6 (12%)	7 (15%)
Stage IV		3 (6%)	1 (2%)

Data are n (%). Staging is based on the 7th edition of TNM (tumour, node, metastasis) staging system for lung cancer.

**Table 3:** Secondary outcomes

	**Conventional diagnosis and staging (n=66)**	**Endobronchial ultrasound-guided transbronchial needle aspiration (n=66)**	**p value**
Investigations per patient	2·39 (0·78)	1·70 (0·72)	<0·0001
Patients diagnosed and staged with one investigation	8 (12%)	30 (45%)	<0·0001
Avoidable thoracotomies at 1 year	13 (76%)	5 (29%)	0·035

Data are mean (SD) or n (%).

## Panel Research in context

**Systematic review**

Before the trial started, we did a review of the scientific literature that was subsequently published.^14^ We compiled the information for this review by searching the PubMed and Medline databases for English-language articles published between Jan 1, 2000, and Jan 1, 2008. Electronic early-release publications were also included. The following search terms were used: “endobronchial ultrasound”, “endoscopic ultrasound”, “lung cancer staging”, “mediastinoscopy”, “positron emission tomography”, “PET-CT”, and “mediastinal staging”. Full articles were obtained and references were checked for additional material, as appropriate. References were chosen on the basis of the highest quality clinical evidence. No randomised trials of patients with suspected lung cancer undergoing EBUS-TBNA only were identified.

**Interpretation**

EBUS-TBNA is recommended for the mediastinal staging of non-small-cell lung cancer and as an alternative to mediastinoscopy. The results from our trial show that routine use of EBUS-TBNA halved the amount of time between testing and treatment decision (compared with conventional diagnosis and staging) and should be considered as the initial investigation after a CT scan for patients with suspected lung cancer and intrathoracic disease at presentation. EBUS-TBNA has a high diagnostic sensitivity for detecting nodal metastases in patients with lung cancer and could reduce the time to treatment decision at no additional cost.
