# Supplementary material for: Lung cancer diagnosis and staging with endobronchial ultrasound-guided transbronchial needle aspiration compared with conventional approaches: an open-label, pragmatic, randomised controlled trial
Source: Lancet Respir Med. 2015 Apr;3(4):282–9. doi: 10.1016/S2213-2600(15)00029-6 (PMC4648022; doi:10.1016/S2213-2600(15)00029-6)
Supplement: Supplementary appendix [file mmc1.pdf]

THE LANCET Respiratory Medicine

Supplementary appendix

This appendix formed part of the original submission and has been peer reviewed. We post it as supplied by the authors.

Supplement to: Navani N, Nankivell M, Lawrence DR, et al, on behalf of the Lung-BOOST trial investigators. Lung cancer diagnosis and staging with endobronchial ultrasound-guided transbronchial needle aspiration compared with conventional approaches: an open-label, pragmatic, randomised controlled trial. *Lancet Respir Med* 2015; published online Feb 4. http://dx.doi.org/10.1016/S2213-2600(15)00029-6.

Appendix

Lung-BOOST trial

Navani et al.

Figure S1: **Conventional diagnosis and staging arm flowchart**

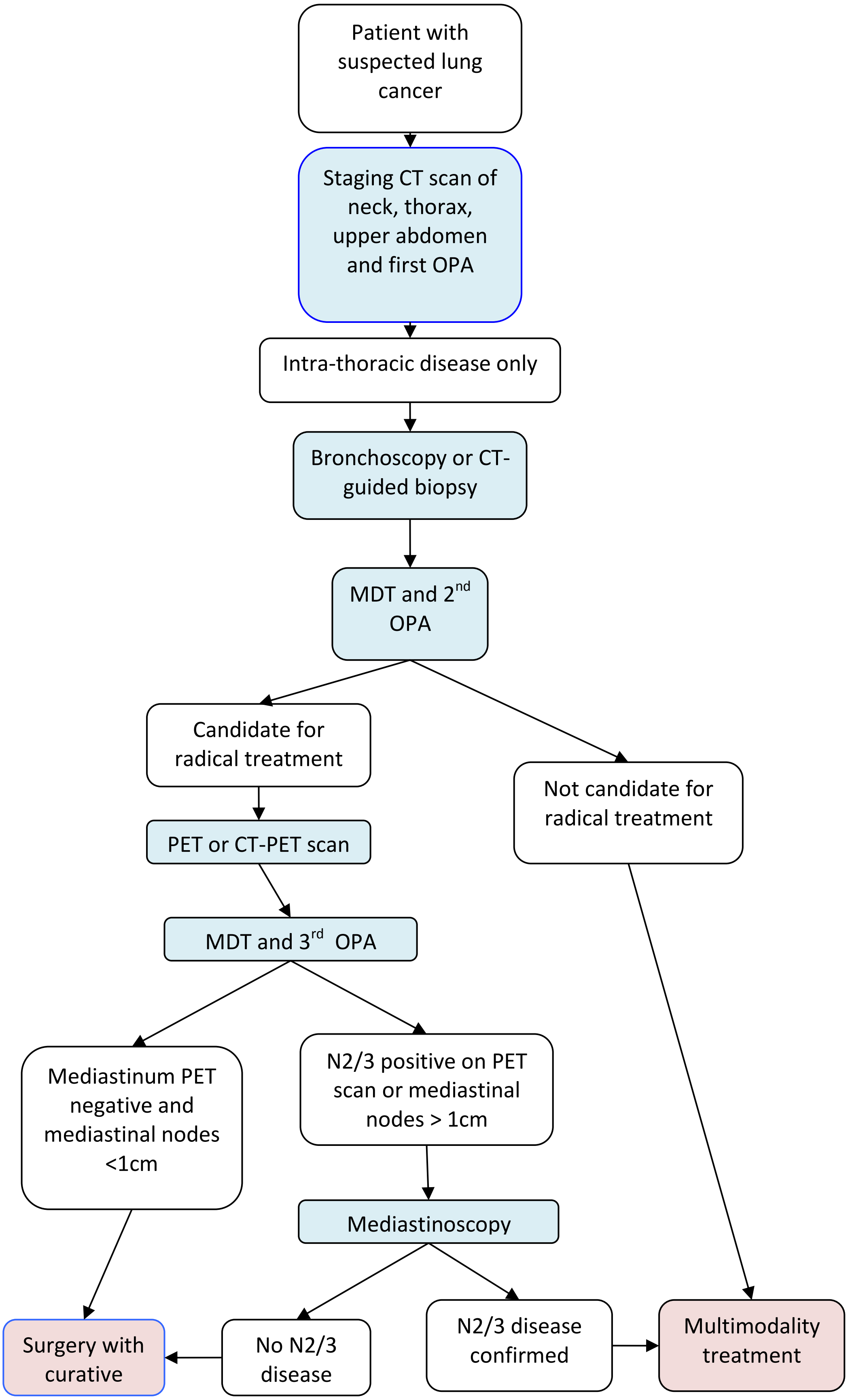

Patients in the conventional diagnosis and staging arm of the trial were recommended to undergo CT guided biopsy or bronchoscopy depending upon whether the primary lesion was peripheral or central. Conventional transbronchial needle aspiration was utilised at the operator’s discretion. If the patient was a candidate for radical treatment, a PET-CT scan was recommended. Mediastinoscopy was advised if the presence of FDG avid lymph nodes precluded a radical treatment option. Invasive mediastinal sampling was also recommended in the trial protocol if any mediastinal lymph node was > 1cm in short axis and its result would alter management. However, the protocol did not mandate any specific investigations (other than the exclusion of EBUS-TBNA) and all investigations and their order, including the need for PET-CT scan and mediastinoscopy, were determined by the multi-disciplinary team.

Figure S2**: EBUS arm flowchart**

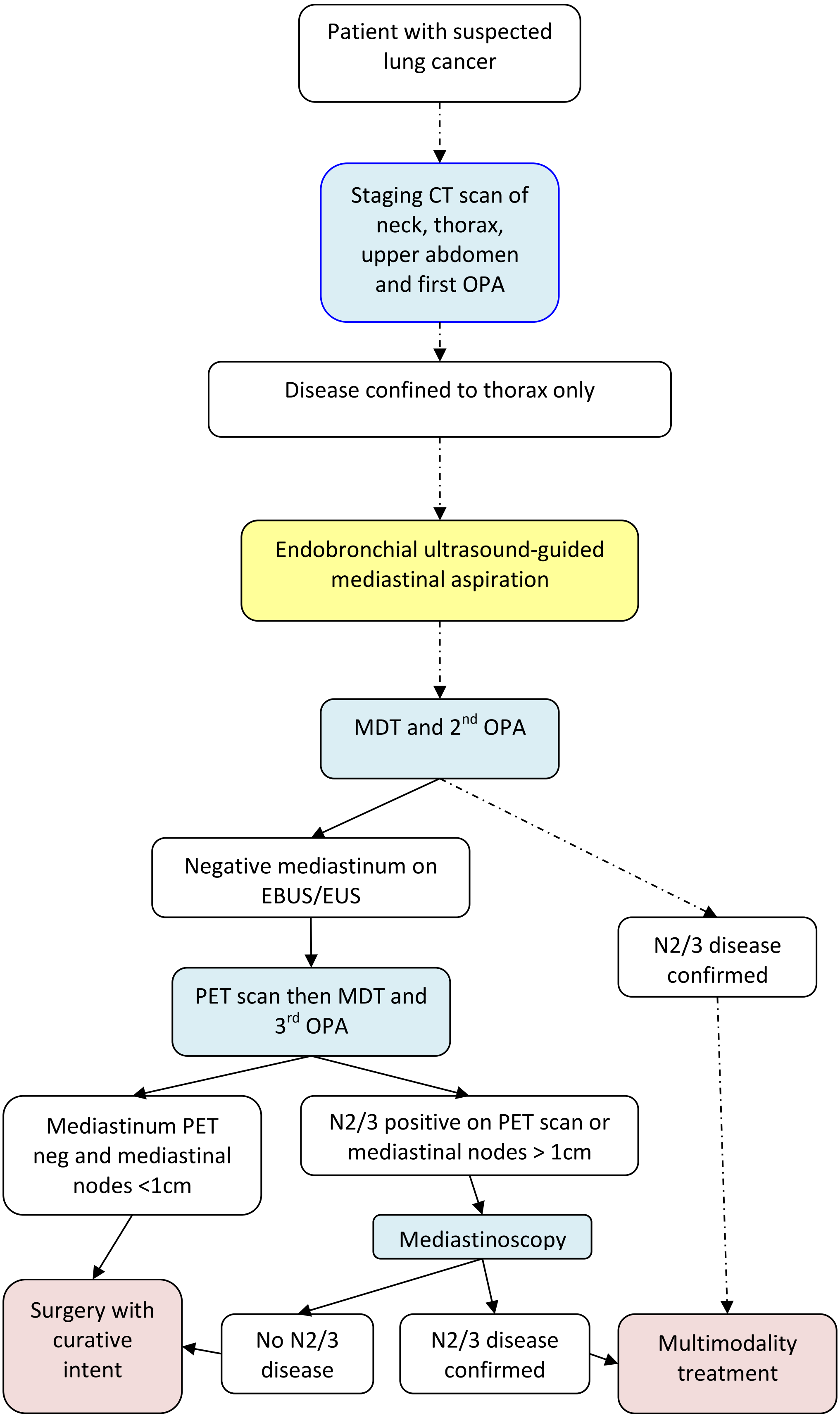

Patients in the EBUS arm of the trial were scheduled to undergo EBUS-TBNA as an initial investigation after staging CT scan. Standard videobronchoscopy was permitted as an additional investigation at the same sitting at the operator's discretion. Endoscopic ultrasound guided fine needle aspiration (EUS-FNA) was permitted as an alternative to EBUS-TBNA if a target lesion was not amenable to EBUS-TBNA. Three to 5 passes per lymph node were made. Rapid on-site evaluation of samples was not performed. Specimens obtained were smeared onto slides and also spun down for cell block analysis. Any cores obtained were transferred directly into formalin and subsequent histopathological examination. Samples from EBUS-TBNA underwent routine laboratory processing. Results from EBUS-TBNA were discussed in multi-disciplinary team meetings in the referring hospitals and further investigations were requested as required.

Figure S3: Lung-BOOST trial CONSORT diagram

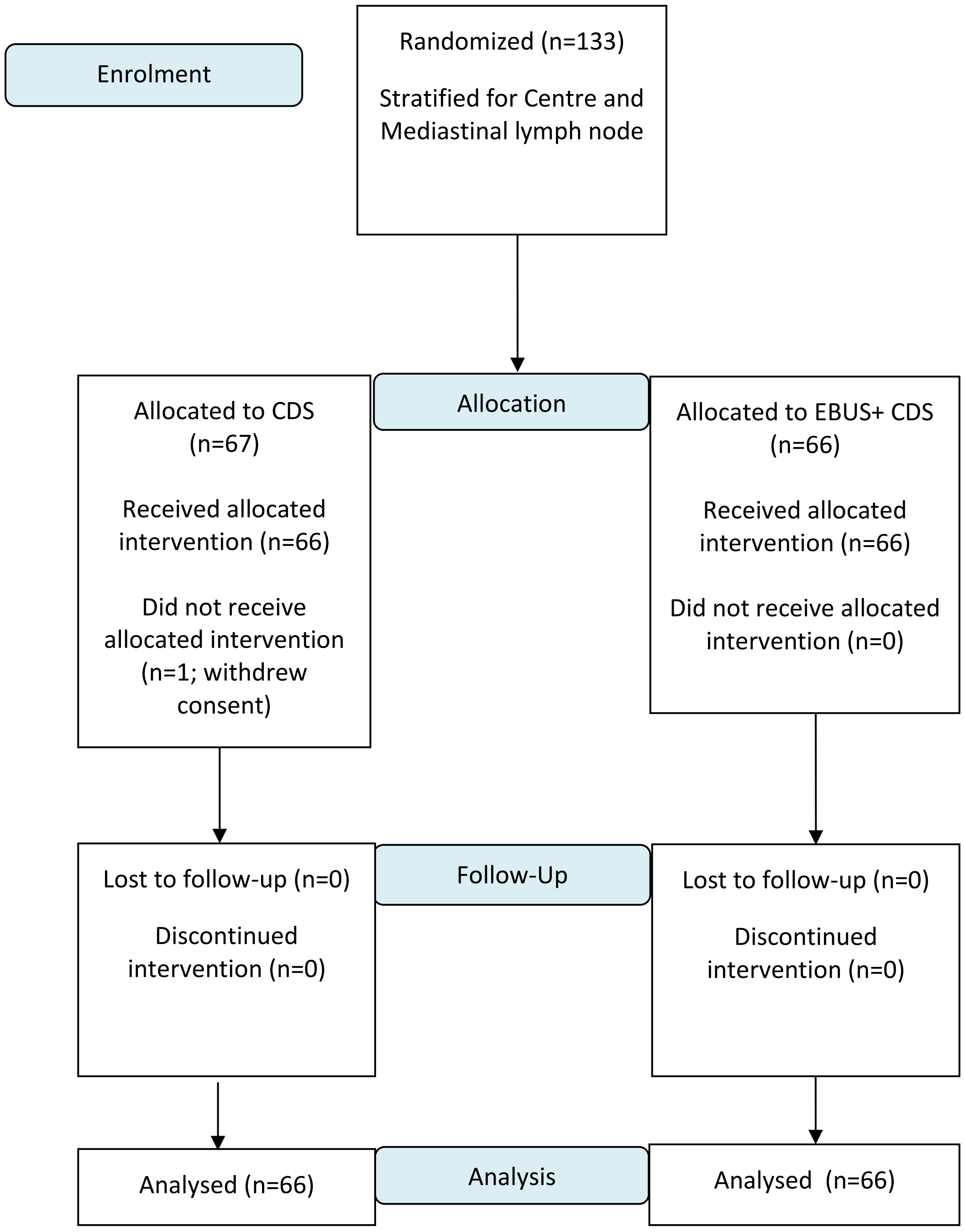

Figure S4: **Exploratory analysis of non-small cell lung cancer survival according to treatment modality in the Lung-BOOST trial**.

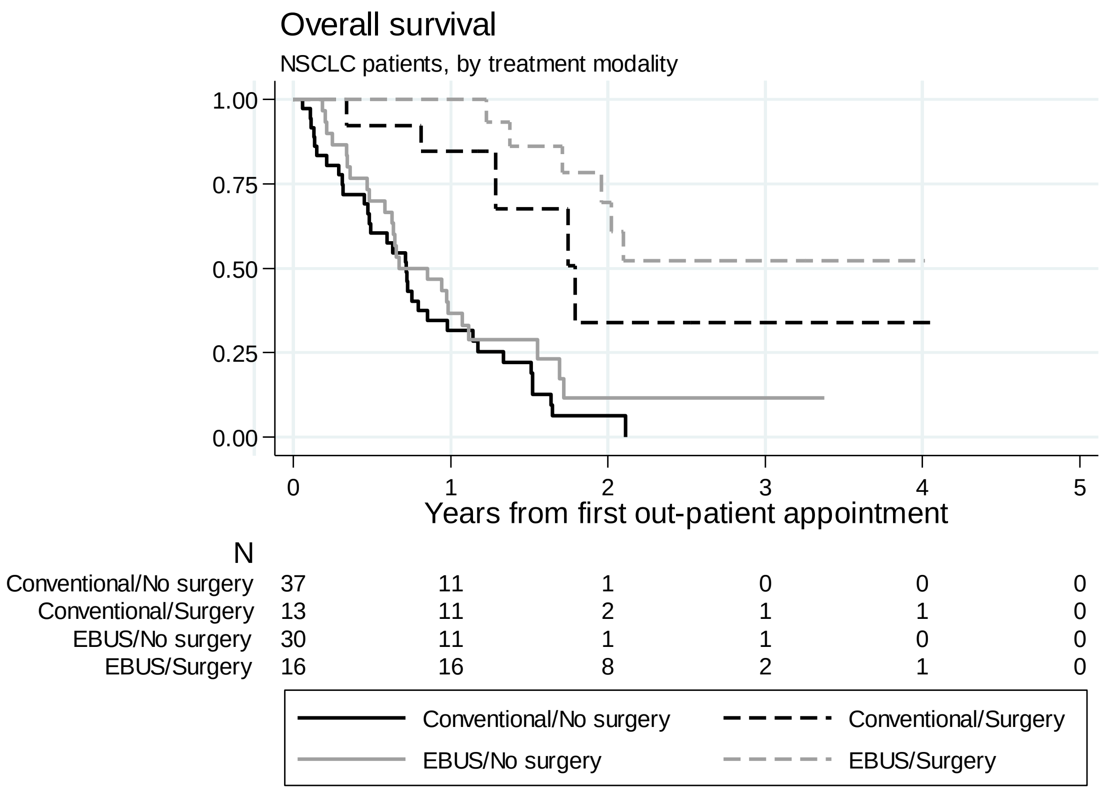

As expected, survival for patients with Stage I and II non-small cell lung cancer undergoing surgery is superior to those patients with more advanced stages not undergoing surgery. However, in patients undergoing surgery there is a trend towards improved survival in patients who have pre-operative EBUS compared to those patients who undergo conventional diagnosis and staging (HR 0.37 (0.10, 1.32), P=0.125). There also may be improved survival in patients undergoing EBUS followed by non-surgical treatments compared to patients undergoing conventional techniques followed by non-surgical treatments (HR 0.68 (0.41, 1.13), P=0.137). These potential differences in survival may reflect a more accurate staging resulting in patients receiving more appropriate and therefore effective therapy for their disease stage.

Table S1: Unnecessary thoracotomies

	Conventional diagnosis and staging (n=17 underwent thoracotomy)	EBUS-TBNA diagnosis and staging (n= 17 underwent thoracotomy)
Unnecessary thoracotomies	13 (76%)	5 (29%)
Open and shut	3	0
Unexpected pN2 or pN3	0	1
Unexpected pT4 or pM1a or b	3	1
Benign disease	3	2
Disease recurrence within 1 year	6	3
Death within 1 year	1	0

Table S2: Costs of diagnosing and staging patients with suspected lung cancer (all patients). Unit costs were obtained from NHS Reference Costs, National Institute of Clinical Excellence 2011 Lung cancer guideline and a published study (9).

	Percentage of patients		Difference	Unit cost (£)	Mean cost per patient		Difference
Procedure	EBUS arm (N=66)	CDS arm (N=66)	p-value		EBUS arm (£)	CDS arm (£)	(£)
Bronchoscopy	3%	67%	<0.001	589	17.85	392.67	-374.82
Conventional TBNA	0%	8%	0.023	162	0.00	12.27	-12.27
Radiology guided biopsy	8%	44%	<0.001	450	34.09	197.73	-163.64
PET-CT scan	50%	76%	0.002	843	421.50	638.64	-217.14
Mediastinoscopy	11%	12%	0.784	3868	410.24	468.85	-58.61
Bone scan	0%	3%	0.154	364	0.00	11.03	-11.03
EBUS	95%	8%	<0.001	1382	1319.18	104.70	1214.48
EUS	5%	3%	0.648	800	36.36	24.24	12.12
Chest drain	2%	2%	1.000	275	4.17	4.17	0.00
Other investigations	0%	13%	<0.001				
VATS	0%	5%		3868	0.00	175.82	-175.82
US liver	0%	5%		64	0.00	2.91	-2.91
MRI head	0%	3%		216	0.00	6.55	-6.55

	Percentage of patients		Difference	Unit cost (£)	Mean cost per patient		Difference
Procedure	EBUS arm (N=66)	CDS arm (N=66)	p-value		EBUS arm (£)	CDS arm (£)	(£)
CT brain	0%	3%		101	0.00	3.06	-3.06
MRI spine	0%	2%		303	0.00	4.59	-4.59
Repeat Bronchoscopy	0%	2%		589	0.00	8.92	-8.92
Repeat Biopsy	0%	2%		450	0.00	6.82	-6.82
Total outpatient appointments (after initial)	103	178	<0.001	100	156.06	279.70	-113.64
Average excess inpatient days	0.03	0.06	0.559	260	7.88	15.76	-7.88
**Total (diagnosis and staging)**					**2407.33**	**2348.41**	**58.92 (95% CI -463, 581)**

Table S3: Costs of initial treatment and overall costs of patients with non-small cell lung cancer. Unit costs were obtained from NHS Reference Costs and NICE 2011 Lung cancer guideline.

	Percentage of patients		Unit cost (£)	Mean cost per patient		Difference
Procedure	EBUS arm (N=46)	CDS arm (N=49)		EBUS arm (£)	CDS arm (£)	(£)
Platinum doublet chemotherapy	46%	16%	4100	1871.74	669.39	1202.35
Lobectomy	35%	27%	5704	1984.00	1513.31	470.69
Radical radiotherapy	4%	14%	2840	123.48	405.71	-282.24
Chemo-radiation	2%	10%	8770	190.65	894.90	-704.25
Palliative radiotherapy	7%	22%	1940	126.52	435.51	-308.99
Palliative care	7%	10%	3581	233.54	365.41	-131.86
**Total (treatment)**				**4530**	**4284**	**246 (-458, 949)**
**Total (diagnosis, staging and treatment)**				**6878**	**6806**	**72 (-925, 1068)**

Pathological staging of patients with non-small cell lung cancer undergoing surgery

Pathological stage	Conventional diagnosis and staging (n=13)	EBUS-TBNA diagnosis and staging (n= 15)
pT1a N0	2	2
pT1a N1		
pT1a N2		
pT1b N0		2
PT1b N1	1	1
pT1b N2		
pT2a N0	1	4
pT2a N1	3	1
pT2a N2		1
pT2b N0		2
pT2b N1		
pT2b N2		
pT3 N0	2	1
pT3 N1	1	
pT3 N2		
pT4 N0	3	1
pT4 N1		
pT4 N2		

Recruitment to Lung-BOOST

	From April 2008	2009	2010	Until July 2011
University College London Hospital	10	27	17	9
North Middx Hospital	10	17	6	3
Whittington Hospital		4	13	7
Princess Alexandra Hospital				2
Nottingham Hospital			1	6
Barnet Hospital			1	
